# Supplementary material for: Effect of a Mismatched Vaccine against the Outbreak of a Novel FMD Strain in a Pig Population
Source: Animals (Basel). 2023 Oct 2;13(19):3082. doi: 10.3390/ani13193082 (PMC10571925; doi:10.3390/ani13193082)
Supplement: Supplementary file 1 [file animals-13-03082-s001.zip › animals-2583547-supplementary.pdf]

Supplementary material

Description of extracted record

The figure below illustrates the accumulative proportion of nursery pigs that developed clinical FMD between 6th and 16th December 2014. The total number of nursery pigs in barn 1, 2, 3, and 4 was 825, 822, 854, and 413, respectively. On 6th December 2014, 16 nursery pigs were randomly blood sampled from barn 2, 3, 4 (78 pigs were sampled from barn 1) for FMD SP ELISA. Among them, 31, 7, 13, and 9 pigs were positive to the ELISA for barn 1, 2, 3, and 4, respectively.

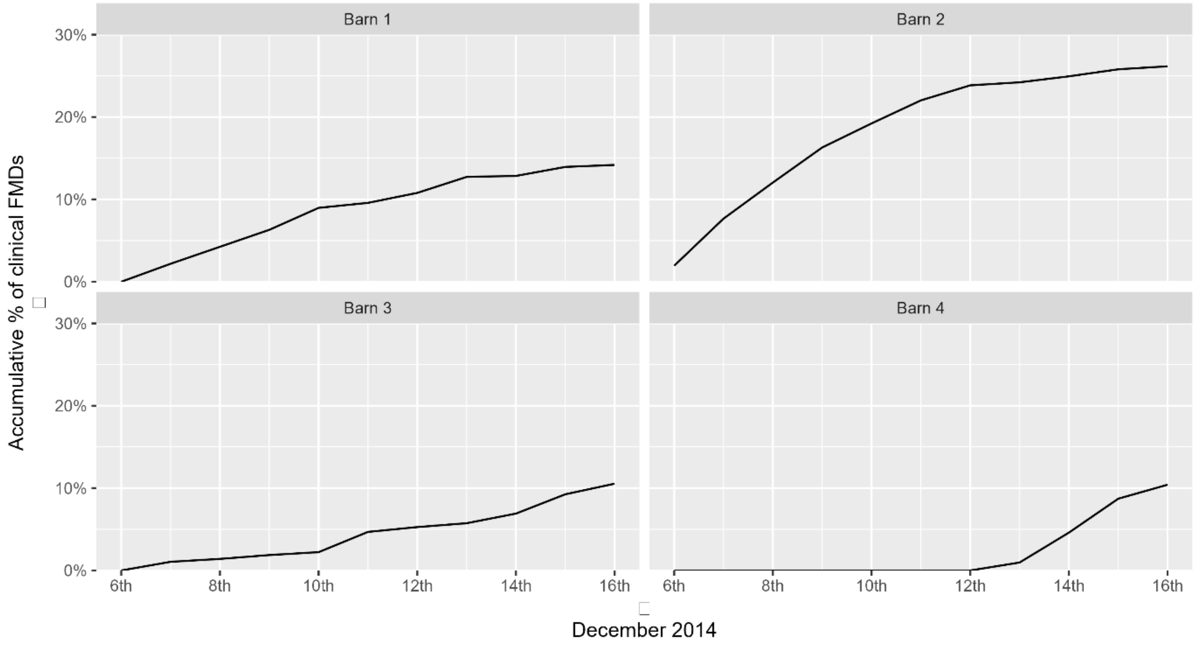

Figure S1. The accumulative proportion of nursery pigs that developed clinical FMD between 6th and 16th December 2014 from a commercial farm in Jinchoen, South Korea.

Model description

Null effect ($\mathcal{M}_1$)

In $\mathcal{M}_1$ (Figure S2), it was assumed that there was no effect of FMD vaccination against the transmission of the novel strain. In the model, nursery pigs were categorised into either susceptible (S), exposed (E), sub-clinically infectious (Is), or clinically infectious (Ic) depending on the infection stage. The novel virus strain was introduced to barn *i* by converting one random susceptible pig in the barn to the E status at Day τ_i during the simulation period. For each susceptible pig in a nursery pen *j* in barn i, the probability of FMD infection (P_{inf}) was estimated as;

$$P_{inf} = 1 - e^{-\lambda}$$

$$\lambda = \beta I_s^j + \beta\omega \sum_{k \neq j} I_s^k$$

where λ is the force of infection, β is the within-pen transmission rate during sub-clinically infectious period (γ_2), k indicates an adjacent pen of pen *j*, ω is the relative decrease in β to adjust for the between-pen transmission, I_s^j and I_s^k are the number of sub-clinically infectious pigs in pen *j* and k in nursery barn i. Exposed pigs (E) became sub-clinically infectious (Is) after the latent period (γ_1), and the animals developed clinical signs and transited from the Is to Ic status after γ_2 days. We assumed that the pigs were removed from the barn on the day the animals transited to the Ic status in the model to mimic the observed record. Due to the lack of exactly how many pigs were in each pen of a certain barn, it was assumed that up to 50 pigs were kept in a pen based on the survey to the farm owner.

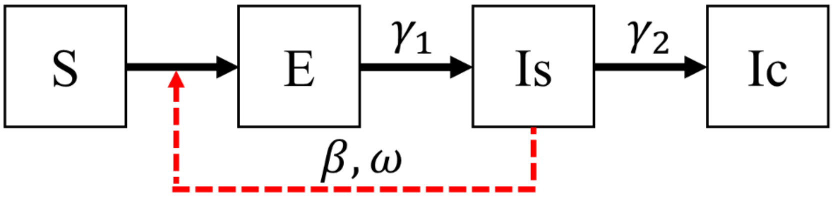

Figure S2. Illustration of FMD compartment in $\mathcal{M}_1$.

Reduced development of clinical FMD ($\mathcal{M}_2$)

Additional features were added on $\mathcal{M}_1$ to establish $\mathcal{M}_2$ that incorporating the disease dynamics for immunised nursery pigs (Figure S3). In the model, immunised nursery pigs were assumed to be susceptible (Sm; immunised susceptible) to the novel strain as well. In the model, a proportion of pigs was assigned to the Sm status for each barn with the proportion for barn *i* (ρ_i) was based on the result of FMD SP antibody ELISA conducted on Day 3. Exposed immunised pigs (Em) became sub-clinical (Ism) after the latent period (γ_1), and then transited to Icm status after γ_2 days, by when they were removed from the barn. The probability of FMD infection (P_{inf}) for a nursery pig in the S or Sm status (pen *j* in barn *i*) was;

$$P_{inf} = 1 - e^{-\lambda}$$

$$\lambda = \beta(I_s^j + I_{sm}^j) + \beta\omega \sum_{k \neq j} (I_s^k + I_{sm}^k)$$

It has been experimentally demonstrated that pigs vaccinated with one strain of FMD virus and later challenged to another strain could shed the virus without developing any clinical signs (Parida et al 2007). Therefore, we established $\mathcal{M}_2$ by assuming that a proportion (α) of immunised but sub-clinically infectious pigs could recover (R) without being transited to the Ic status. Pigs in the Ism status were assumed to be infectious for γ_3 days.

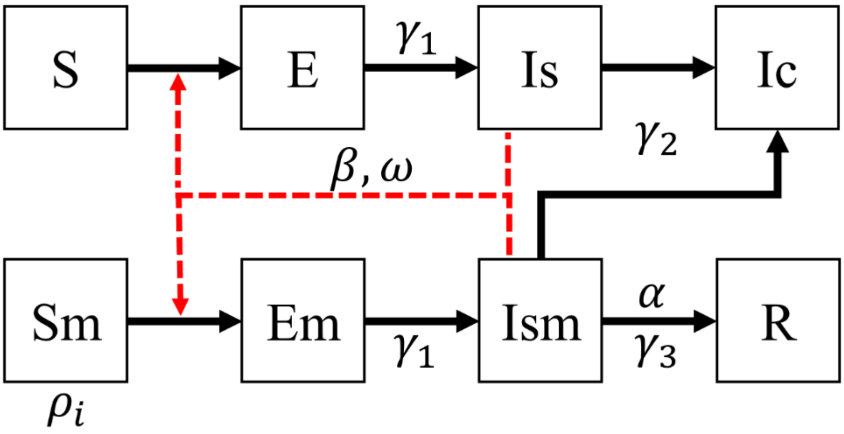

Figure S3. Illustration of FMD compartment in $\mathcal{M}_2$.

Reduced transmission ($\mathcal{M}_3$)

The model $\mathcal{M}_3$ was somewhat similar to $\mathcal{M}_2$, except that immunised but sub-clinically infectious pigs (i) were less likely to transmit the novel strain of FMD virus, and (ii) should develop clinical signs. To simulate the less likeliness of transmission, the within-pen transmission rate for immunised but sub-clinically infectious pigs reduced by φ compared with pigs in the Is status (Figure S4).

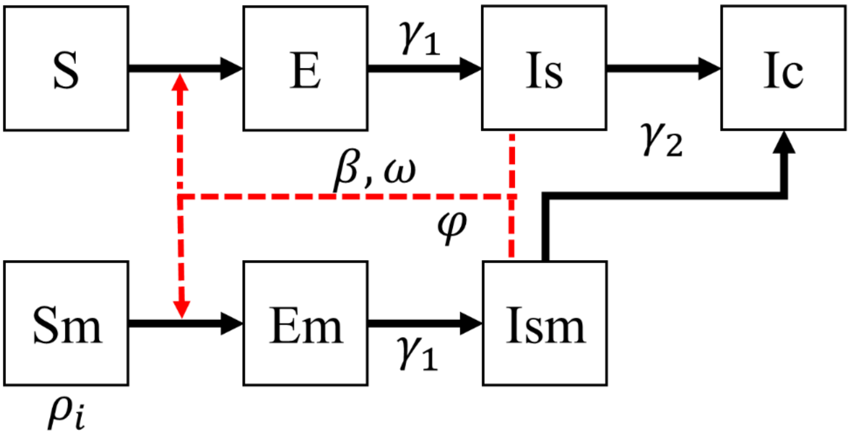

Figure S4. Illustration of FMD compartment in $\mathcal{M}_3$.

The probability of FMD infection (P_{inf}) for a nursery pig in the S or Sm status (pen j in barn i) was;

$$P_{inf} = 1 - e^{-\lambda}$$

$$\lambda = \beta(I_s^j + \varphi I_{sm}^j) + \beta\omega \sum_{k \neq j} (I_s^k + \varphi I_{sm}^k)$$

Reduced transmission and development of clinical FMD ($\mathcal{M}_4$)

In $\mathcal{M}_4$ (Figure S5), reduction in both transmission and development of clinical FMD for immunised but sub-clinically infectious pigs was considered. The formula to calculate the probability of FMD infection (P_{inf}) for a nursery pig in the S or Sm status was identical to the one used in $\mathcal{M}_3$.

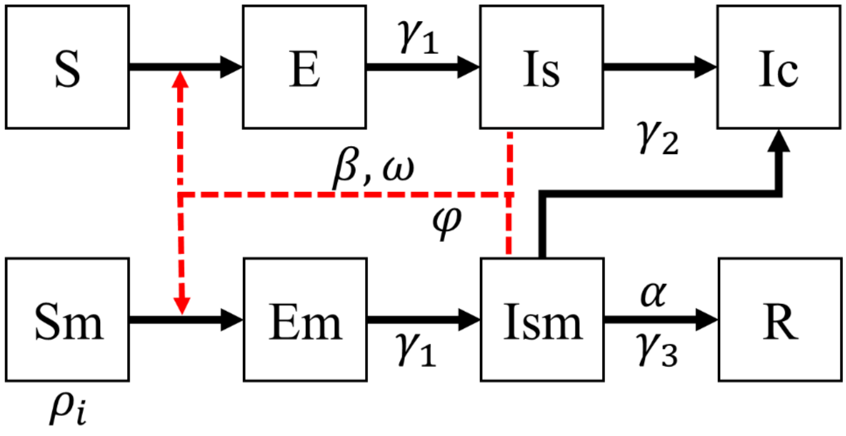

Figure S5. Illustration of FMD compartment in $\mathcal{M}_4$

Full effect ($\mathcal{M}_5$)

In $\mathcal{M}_5$ (Figure S6), a proportion of pigs (ρ_i) was assigned to the M (fully immunised) status, and these pigs were assumed to be fully protected from the infection. The formula to calculate

the probability of FMD infection (P_{inf}) for a nursery pig in the S or Sm status was identical to the one used in $\mathcal{M}_1$.

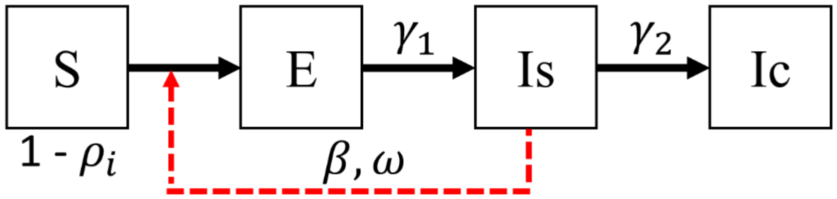

Figure S6. Illustration of FMD compartment in $\mathcal{M}_5$

It is worth noting that neither $\mathcal{M}_1$ nor $\mathcal{M}_5$ can be considered as realistic scenarios, because it is unlikely for immunised pigs to be completely unprotected or fully shielded from the novel strain, respectively. Nevertheless, we included these extreme cases to examine whether they could better explain the observed data.

ABC-SMC algorithm

The detailed ABC-SMC algorithm was as below.

(1) Set t = 1, where t is the SMC sequence indicator. Initialise the threshold values for the first round (t = 1), $\varepsilon_1, \varepsilon_2, \varepsilon_3, \varepsilon_4$ by;

  a. Running $\mathcal{M}_3$ for 500 times without rejecting any particles, and estimating the distances (D_k) of summary statistics as;

$$D_k = \sqrt{\sum_{t=1}^{T} (S_t^k - O_t^k)^2}$$

  where T is the duration that the pigs with clinical signs were removed (11 days), and S_t^k and O_t^k respectively indicate the simulated and observed number of pigs that were removed due to the development of clinical signs at Day t from barn k.

  b. Setting $\varepsilon_1, \varepsilon_2, \varepsilon_3, \varepsilon_4$ as the median values of D_1, D_2, D_3, D_4 respectively.

(2) Set i = 1, where i is the particle indicator.

(3) Generate a particle of parameter set, θ, by;

  a. If t = 1, sample θ^{**} from $\pi(\theta)$, where $\pi(\theta)$ is the prior distributions of θ.

  b. If t > 1, sample θ^{*} from the particles of previous sequence, $\{\theta_{t-1}\}$, with weights, $\{w_{t-1}\}$. Then perturb the particle $\theta^{**} \sim K(\theta|\theta^{*})$, where $K(\cdot)$ is a perturbation kernel. In this study, we used a component-wise Gaussian kernel with the variance as two times of the variance of particles in the previous SMC sequence. If the probability of $\pi(\theta^{**})$ equals 0, return to (3).

(4) Run $\mathcal{M}_3$ for each barn with the generated particles and calculated D_k.

(5) Accept θ^{**} as the particle θ_t^i if $D_1 < \varepsilon_1$, $D_2 < \varepsilon_2$, $D_3 < \varepsilon_3$, and $D_4 < \varepsilon_4$ otherwise return to (3).

(6) Calculate weight for the particle, w_t^i, as;

a. If t = 1, $w_t^i = 1$,

b. If t > 1, $w_t^i = \pi(\theta_t^i)/\sum_{j=1}^{N} w_{t-1}^{j} K(\theta_t^i, \theta_{t-1}^{j})$.

(7) Set i = i + 1, and repeat (3) ~ (6) until i = 500. It indicates that 500 particles for each parameter were accepted for one SMC sequence.

(8) Normalise the weights (divide the weight by the sum of weight) and calculate the effective sample size (ESS) as;

$$ESS = \frac{1}{\sum_1^{500}(\bar{w}^i)^2}$$

where $\bar{w}^i$ was normalised weight.

(9) Update $\varepsilon_1, \varepsilon_2, \varepsilon_3, \varepsilon_4$ to the median values of D_1, D_2, D_3, D_4, respectively. Set t = t + 1. Return to (1) until t = 12.

In total, we sampled approximately 63.1 million particles and ESS of any parameter during the whole sequence was between 77 and 498.

Posterior distribution

In $\mathcal{M}_3$, there were 13 parameters to be estimated ($\beta, \gamma_1, \gamma_2, \varphi, \omega, \rho_1, \rho_2, \rho_3, \rho_4, \tau_1, \tau_2, \tau_3, \tau_4$). The estimated parameter values are schematised in Figure S7 ~ S11 with the median and 95% credible interval of the values being provided in Table S1.

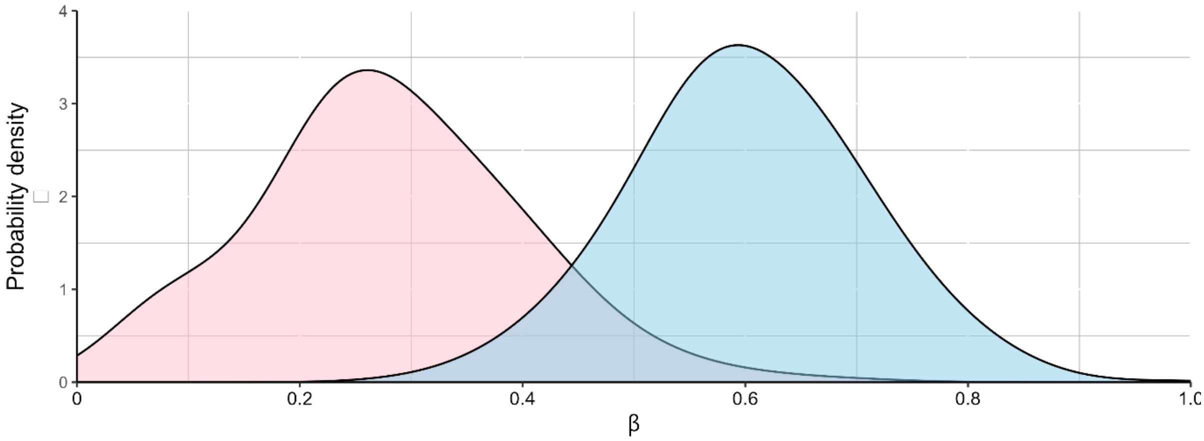

Figure S7. The probability density of the posterior distribution (pink) of within-pen transmission rate (β). The blue area illustrates the prior distribution.

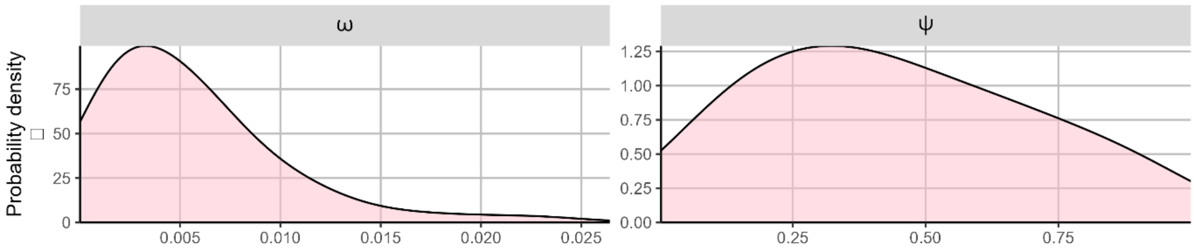

Figure S8. The probability density of the posterior distribution of the relative decrease of β for between-pen transmission (ω) and relative decrease in β for immunised pigs (φ).

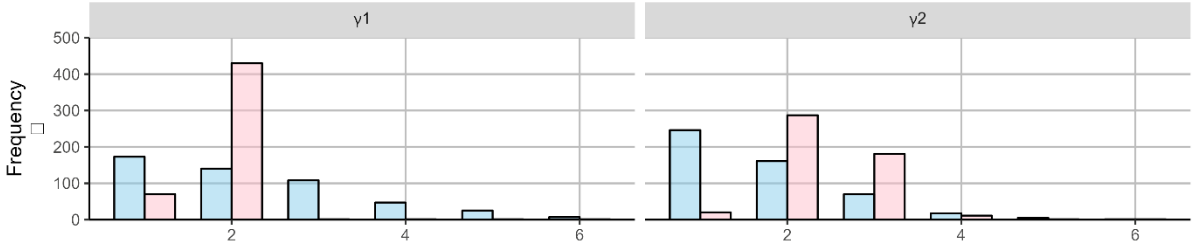

Figure S9. The frequency of the posterior distribution (pink) of the latent period (γ_1; left) and sub-clinically infectious period before developing clinical signs (γ_2; right). The blue area illustrates the prior distributions.

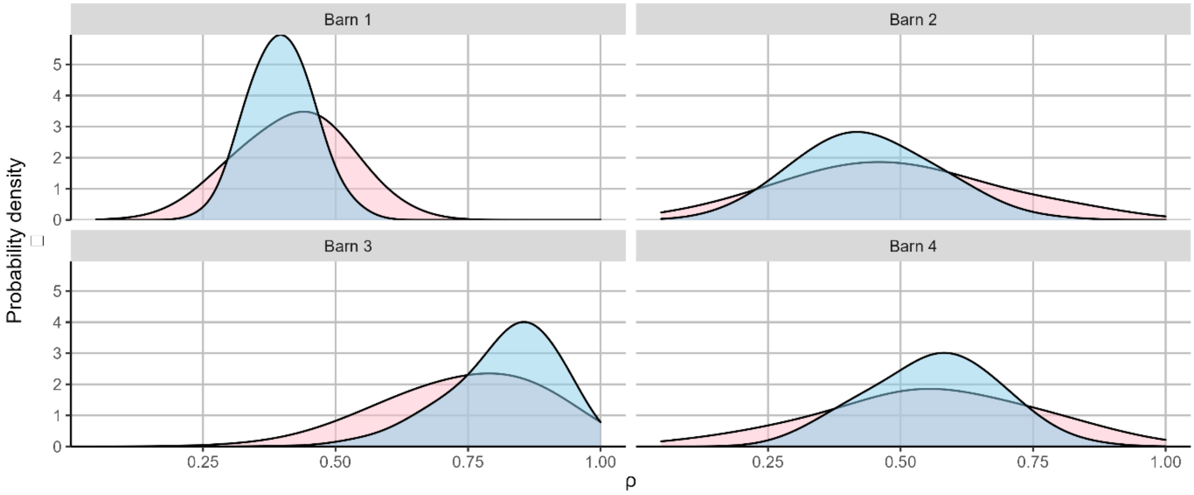

Figure S10. The probability density of the posterior distribution (pink) of the proportion of immunised pigs for each barn (ρ). The blue area illustrates the prior distribution.

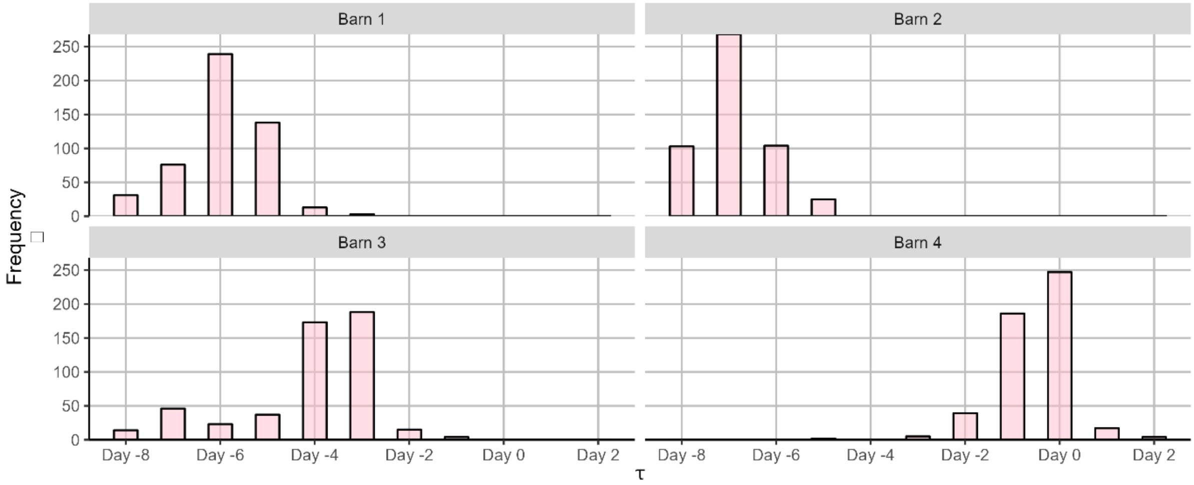

Figure S11. The frequency of the posterior distribution of the day of novel FMD virus introduction for each barn (τ).

Table S1. The posterior distribution of all the estimated parameters in $\mathcal{M}_3$.

**Parameter**	**Median (95% credible interval)**
Within-pen transmission rate (β)	0.269 (0.066, 0.524)
Relative decrease in β for between-pen transmission (ω)	0.004 (0.000, 0.019)
Latent period (γ_1)	2 (1, 2) days
Sub-clinically infectious period before developing clinical signs (γ_2)	2 (1, 3) days
Relative decrease in β for immunised pigs (φ)	0.401 (0.031, 0.941)
Proportion of immunised pigs – barn 1 (ρ_1)	0.426 (0.223, 0.607)
Proportion of immunised pigs – barn 2 (ρ_2)	0.474 (0.127, 0.860)
Proportion of immunised pigs – barn 3 (ρ_3)	0.760 (0.442, 0.975)
Proportion of immunised pigs – barn 4 (ρ_4)	0.554 (0.137, 0.909)
Day of novel FMD virus introduction – barn 1 (τ_1)	Day -6 (-8, -4)
Day of novel FMD virus introduction – barn 2 (τ_2)	Day -7 (-8, -5)
Day of novel FMD virus introduction – barn 3 (τ_3)	Day -4(-8, -2)
Day of novel FMD virus introduction – barn 4 (τ_4)	Day 0 (-2, 1)

Sensitivity analysis

Effect of different parameters

The result of sensitivity analysis on the effect of different parameters on the summary statistics and cumulative number of pigs with clinical FMD is provided in Table S2. The sensitivity analysis showed that, in $\mathcal{M}_3$, both the summary statistics and accumulative number of clinical FMD were most affected by the day of novel FMD virus introduction (τ), followed by the latent period (γ_1). The next most affecting parameter was the sub-clinically infectious period (γ_2) for the summary statistics, whereas it was the number of pigs initially exposed to the novel FMD strain for the accumulative number. This result indicated that the prior information about $\gamma_1$and γ_2 was important in identifying other parameter values in $\mathcal{M}_3$.

Table S2. Result of partial rank correlation coefficients for the summary statistics and accumulative number of pigs with clinical FMD in $\mathcal{M}_3$. Please note that the presented result below was based on the analysis on barn 1, however, the result was similar across other barns.

**Parameters**	**Summary statistics**	**Accumulative clinical FMD**
Within-pen transmission rate (β)	0.11	0.13
Relative decrease in β for between-pen transmission (ω)	0.08	0.06
Latent period (γ_1)	-0.57	-0.34
Sub-clinically infectious period before developing clinical signs (γ_2)	-0.42	-0.11
Relative decrease in β for immunised pigs (φ)	0.03	0.05
Proportion of immunised pigs (ρ_1)	-0.04	-0.03
Day of novel FMD virus introduction (τ_1)	-0.92	-0.42
Number of pigs initially exposed to the novel FMD strain	0.29	0.14

Effect of different priors

The posterior distributions of the parameter based on a uniform prior of $\beta, \gamma_1, \gamma_2$ are provided in Table S3. Compared with the results using an informed prior, the posterior distributions with a uniform prior of either β or γ_1 were not remarkably different except slightly increased within-pen transmission rate value. Interestingly, the posterior distributions of most parameters except (ρ_i) were highly affected by a uniform prior of γ_2, indicating that the identifiability of the model highly depends on the information about the sub-clinically infectious period.

Table S3. The posterior distribution of all the estimated parameters in $\mathcal{M}_3$ when informed priors (Original), and a uniform prior of β, γ_1, γ_2 were used.

**Parameters**	**Original**	**Uniform $\boldsymbol{\beta}$**	**Uniform $\boldsymbol{\gamma_1}$**	**Uniform $\boldsymbol{\gamma_2}$**
β	0.269 (0.066, 0.524)	0.368 (0.223, 0.486)	0.367 (0.110, 0.546)	0.527 (0.188, 0.870)
ω	0.004 (0.000, 0.019)	0.002 (0.000, 0.007)	0.003 (0.000, 0.009)	0.182 (0.008, 0.486)
γ_1	2 (1, 2) days	2 (1, 2) days	2 (1, 2) days	3 (1, 6) days
γ_2	2 (1, 3) days	3 (2, 3) days	3 (2, 4) days	4 (1, 7) days
φ	0.401 (0.031, 0.941)	0.403 (0.017, 0.939)	0.340 (0.019, 0.898)	0.494 (0.018, 0.955)
ρ_1	0.426 (0.223, 0.607)	0.417 (0.234, 0.603)	0.411 (0.215, 0.597)	0.392 (0.222, 0.554)
ρ_2	0.474 (0.127, 0.860)	0.524 (0.108, 0.909)	0.528 (0.103, 0.890)	0.445 (0.081, 0.870)
ρ_3	0.760 (0.442, 0.975)	0.754 (0.429, 0.986)	0.752 (0.368, 0.971)	0.795 (0.415, 0.981)
ρ_4	0.554 (0.137, 0.909)	0.551 (0.169, 0.915)	0.573 (0.171, 0.906)	0.565 (0.182, 0.916)
τ_1	Day -6 (-8, -4)	Day -6 (-8, -5)	Day -6 (-8, -5)	Day 5 (-4, 11)
τ_2	Day -7 (-8, -5)	Day -8 (-8, -6)	Day -7 (-8, -6)	Day 5 (-4, 10)
τ_3	Day -4(-8, -2)	Day -4 (-8, -1)	Day -4 (-8, -2)	Day 0 (-7, 5)
τ_4	Day 0 (-2, 1)	Day 1 (1, 2)	Day 1 (-2, 1)	Day 4 (-4, 11)

β ~ Uniform(0.0, 1.5)

γ_1 ~ Uniform(1, 7) conditional on $\gamma_1 + \gamma_2 \leq 9$

γ_2 ~ Uniform(1, 9) conditional on $\gamma_1 + \gamma_2 \leq 9$
